# Developmental changes in analytic and holistic processes in face perception

**DOI:** 10.3389/fpsyg.2015.01165

**Published:** 2015-08-07

**Authors:** Jane E. Joseph, Michelle D. DiBartolo, Ramesh S. Bhatt

**Affiliations:** ^1^Department of Neurosciences, Medical University of South Carolina, Charleston, SCUSA; ^2^Department of Psychology, University of Kentucky, Lexington, KYUSA

**Keywords:** holistic, configural, featural, similarity, face inversion, children, perceptual matching, serial, parallel

## Abstract

Although infants demonstrate sensitivity to some kinds of perceptual information in faces, many face capacities continue to develop throughout childhood. One debate is the degree to which children perceive faces analytically versus holistically and how these processes undergo developmental change. In the present study, school-aged children and adults performed a perceptual matching task with upright and inverted face and house pairs that varied in similarity of featural or 2^nd^ order configural information. Holistic processing was operationalized as the degree of serial processing when discriminating faces and houses [i.e., increased reaction time (RT), as more features or spacing relations were shared between stimuli]. Analytical processing was operationalized as the degree of parallel processing (or no change in RT as a function of greater similarity of features or spatial relations). Adults showed the most evidence for holistic processing (most strongly for 2^nd^ order faces) and holistic processing was weaker for inverted faces and houses. Younger children (6–8 years), in contrast, showed analytical processing across all experimental manipulations. Older children (9–11 years) showed an intermediate pattern with a trend toward holistic processing of 2^nd^ order faces like adults, but parallel processing in other experimental conditions like younger children. These findings indicate that holistic face representations emerge around 10 years of age. In adults both 2^nd^ order and featural information are incorporated into holistic representations, whereas older children only incorporate 2^nd^ order information. Holistic processing was not evident in younger children. Hence, the development of holistic face representations relies on 2^nd^ order processing initially then incorporates featural information by adulthood.

## Introduction

A wealth of research suggests that face recognition and identification improve with age throughout childhood and adolescence (Goldstein and Chance, 1964; Ellis et al., 1973; Kagan and Klein, 1973; Carey and Diamond, 1977; Carey et al., 1980; Ellis and Flin, 1990; Pascalis and Slater, 2003; Gauthier and Nelson, 2001; de Heering et al., 2012). Although numerous perceptual mechanisms have been examined, there continues to be debate as to which mechanism(s) are most critical for the proficient and expert-level face recognition demonstrated by adults. *Configural* processing refers to processing the spatial relations among facial features, with *1^st^ order* configuration referring to the canonical ordering of facial features in an upright orientation (eyes above nose above mouth) and *2^nd^ order* configuration referring to the spacing of the features relative to each other. *Holistic* processing refers to perceiving the individual features and their spatial relations as an integrated whole (Pascalis et al., 2011). *Analytical*, *featural*, or *piecemeal* processing of faces refers to perceiving, comparing, or analyzing specific face components, such as the eyes, nose, mouth.

Diamond and Carey (1986) and Carey and Diamond (1994) suggested that perceptual expertise for faces is based on proficiently encoding and using 2^nd^ order information. In their model, objects within a category are compared to a configural prototype in order to discriminate different exemplars. Computing 2^nd^ order information supports rapid and accurate discrimination among the exemplars of the same category. Although faces are the only class of stimuli with which most adults have sufficient expertise to allow the use of 2^nd^ order information (Carey and Diamond, 1994; Tanaka and Farah, 2003; Tarr and Cheng, 2003), the same processing may be used to support expertise with other visual categories (Diamond and Carey, 1986).

Carey and Diamond (1994) also suggested that younger children have not yet developed the perceptual capacity for 2^nd^ order processing of faces and, instead, rely on a featural encoding strategy for identifying upright and inverted faces. They based this conclusion on the finding that 6-year-olds recognized inverted faces as well as upright faces, whereas 8- and 10-year-olds exhibited an inversion effect that is similar to that shown by adults. In other words, older children and adults demonstrate greater difficulty with face identification when faces are inverted but object identification is not as strongly affected (Yin, 1969). One interpretation of the face inversion effect is that configural and holistic processing, which may be more integral to faces than to other objects, is disrupted with inversion so that an inverted face becomes more like a collection of features rather than an integrated, holistic gestalt (Rossion, 2009). Individual features of objects (e.g., the mane of a horse) may be sufficient to uniquely identify an object at a basic-level of categorization, so inversion has little impact on object recognition. In support of this, many studies indicate that inversion affects relational processing more than featural processing (Thompson, 1980; Bartlett and Searcy, 1993; Rhodes et al., 1993; Freire et al., 2000; Murray et al., 2000, 2003; Barton et al., 2001; Le Grand et al., 2001).

This interpretation, however, has been questioned by findings that inversion may also disrupt processing of other non-face categories or face stimuli without internal features (Reed et al., 2003; Brandman and Yovel, 2012). Also, inversion disrupts featural processing of faces in addition to configural processing (Maurer et al., 2002; Riesenhuber et al., 2004; Sekuler et al., 2004). Debates continue about whether featural and configural processing of faces are independent components of face processing (Riesenhuber and Wolff, 2009) and whether featural processing of faces is equivalent to object processing (Peterson and Rhodes, 2003). For example, face inversion effects are much weaker if stimuli are perceptually very similar (Rhodes et al., 2006), and the differential effect of inversion on relational versus featural processing goes away under these conditions. Also, when faces are inverted, participants may use the same local information to discriminate faces, but they do this less efficiently compared with upright faces (Sekuler et al., 2004). Given this debate and the fact that face inversion has been a well used manipulation to study developmental changes in face processing, the present study will examine the effect of inversion on both featural and configural processing across different levels of similarity, in both children and adults.

One reason that face inversion effects have been intensely investigated in developmental studies is that many studies have replicated the findings by Carey and Diamond that younger children show weaker face inversion effects than older children and adults (Schwarzer, 2000; Brace et al., 2001; Joseph et al., 2006; Meinhardt-Injac et al., 2014). Presumably, if children perceive faces as a collection of features rather than as an integrated gestalt, then inversion will not disrupt processing of the individual features. In contrast, inversion will disrupt 2^nd^ order configural processing because spatial relations cannot be as easily perceived when the canonical orientation is changed. Studies that have directly manipulated featural and 2^nd^ order information in faces have also reported earlier development of (and reliance on) featural processing in children, compared to 2^nd^ order processing (Schwarzer, 2000; Freire and Lee, 2001; Maurer et al., 2002; Mondloch et al., 2002, 2003, 2006). This delayed development of 2^nd^ order processing, however, is debated (Gilchrist and McKone, 2003; Pellicano et al., 2006). McKone and Boyer (2006) argued that when baseline performance is accounted for, children as young as 4 years of age show sensitivity to 2^nd^ order information in faces, similar to the sensitivity shown by adults. In addition, developmental delays in 2^nd^ order processing may not be specific to faces (Robbins et al., 2011) suggesting a more domain-general mechanism at play. Although sensitivity to 2^nd^ order information in faces can improve (Baudouin et al., 2010) or become more specific to faces (Cassia et al., 2011) with age, sensitivity to 2^nd^ order information emerges as early as 5 months of age (Hayden et al., 2007). Nevertheless, if younger children (and infants) are sensitive to 2^nd^ order information, then why do they show reduced face inversion effects compared to older children and adults?

**FIGURE 1:**
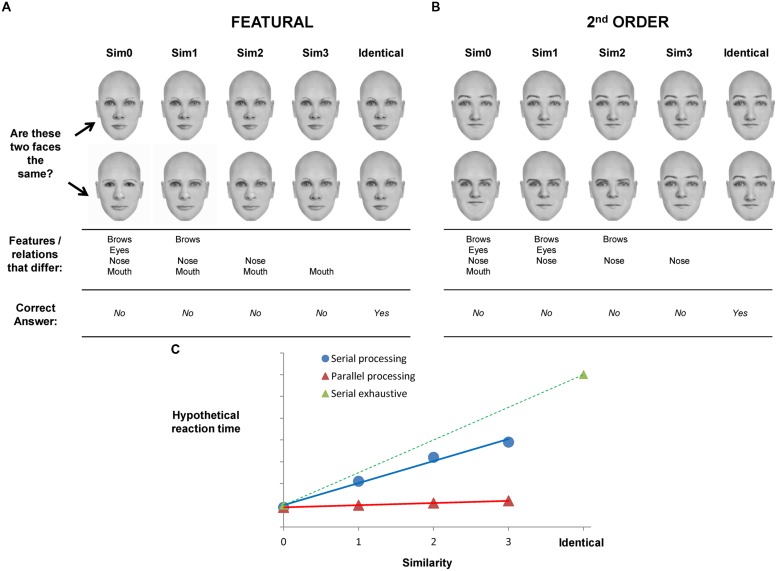
**Illustration of the experimental paradigm and hypotheses of the present study. (A)** Sample featural face pairs (top two rows) that varied across similarity levels. Features that differed for each similarity level are shown in the third row and the fourth row indicates the correct response for a pair **(B)** sample 2^nd^ order face pairs are shown. Third row indicates the spacing relations that were different (distance of brows to top of head, distance of nose to top of head, distance of mouth to top of head and interocular distance). **(C)** Hypothetical reaction time is illustrated on the *y*-axis as a function of degree of similarity. Serial processing is indicated by an increase in RT as a function of similarity (blue line) and parallel processing is indicated by no change in RT as a function of similarity (red line) on sim0–sim3 trials. Identical pairs represent the maximum similarity two faces can share. In this case, holistic representations would lead to a serial exhaustive comparison process (green dotted line).

**FIGURE 2:**
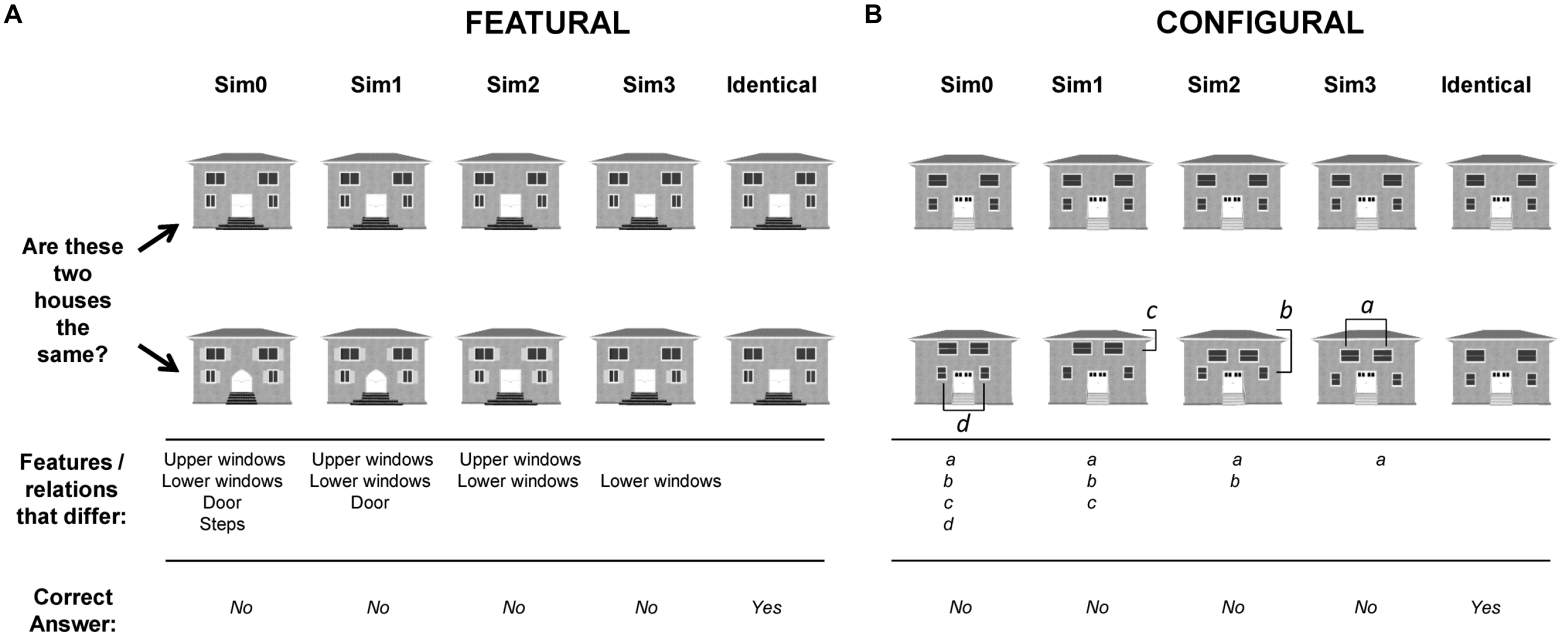
**Illustration of house stimuli used in the study. (A)** Shows house pairs that differed in internal features (upper and lower windows, doors, steps). **(B)** Shows house pairs that differed in spacing distances of some of the features (distance of upper windows to roof, distance of lower windows to roof, distance between upper windows, distance between lower windows). The task and responses were the same as those required for face stimuli.

The goal of the present study is to explore this question further by using a perceptual matching task and parametrically varying featural and 2^nd^ order configural information (**Figures 1** and **2**). Importantly, the same perceptual processing will be examined in another class of objects (houses) which are well equated to the face stimuli in terms of the type of information manipulated and the level of differentiation required. In addition, the analyses will control for performance differences across adults, older children (9–12 years of age) and younger children (6–8 years of age) by using baseline performance as a covariate.

The experimental paradigm is illustrated in **Figure 1**. Two faces (or houses) were presented simultaneously and subjects decided if they were the same or different. If two identical stimuli were presented, the correct response was “yes” (as indicated by a button press). If the stimuli presented were different in any way, then the correct response was “no” (as indicated by a button press). In the featural condition (**Figure 1A**), the “different” pairs consisted of stimuli that differed in 1, 2, 3, or 4 internal features. In the example shown, the “sim0” pairs were different in all features (brows, eyes, nose, mouth); hence, similarity was 0. “Sim1” pairs had three features that were different, “sim2” pairs had two different features and “sim3” pairs differed by only one feature. The actual features that varied at each similarity level were different across pairs so that, for example, not all sim2 pairs differed by the nose and mouth (as shown in the figure); some sim1 pairs varied by the eyes and brows, etc.

Much of the evidence from studies using a similar paradigm of parametric manipulation of similarity (e.g., Collins et al., 2012) has shown that the more features that are similar, the harder it is to reject the two stimuli as the same. Or, the more features that are different, the easier it is to reject the two stimuli as the same. Consequently, reaction time (RT) and/or error rates increase as similarity increases for “different” trials (i.e., trials in which a correct response is “no”). This pattern of results would be expected if the stimuli are compared on a feature-by-feature basis. An increase in RT/errors that is linear and monotonic is often taken as a reflection of serial processing of the features, (blue line in **Figure 1C**), as in the frameworks of visual selective attention (Treisman and Gelade, 1980) or short-term memory search (Sternberg, 1966); that is, each additional level of similarity will incrementally increase RT/errors because the stimuli are compared feature-by-feature until a difference is found. If more features are similar, then more comparisons would be made and RT would be longer. Here, we suggest that if the features of a face are integrated into a holistic percept, then access to individual face features will be more difficult. RT functions will show evidence for serial processing because a feature-by-feature comparison will take longer if more features are similar; that is, more features will need to be compared before finding a difference in features. Hence, if RT functions have a positive slope, then serial processing is assumed and this, in turn, is a reflection of holistic representations.

Conversely, if features can be processed independently, then the number of features that are different on any given trial will not affect performance. This is akin to parallel processing of features, much like the idea of preattentive processing and pop-out effects in visual selective attention (Treisman and Gelade, 1980). Parallel processing means that the individual features can be processed simultaneously so that a feature-by-feature comparison is not necessary. Hence, the ideal parallel processor would show no difference in RT/errors as a function of similarity (red line in **Figure 1C**) because detecting just one different feature is sufficient to make a “no” response and the number of additional different features will not significantly increase RT. We suggest that RT functions that do not have a significant positive slope reflect parallel processing. In turn, this pattern of results reflects underlying face representations that are more piece-meal and not integrated into a holistic percept.

Matching of two identical stimuli may involve a serial exhaustive comparison of all features (dotted green line in **Figure 1C**) if the features are integrated into a holistic percept and cannot be processed or analyzed independently. “Same” trials are depicted (and will be analyzed) separately from “different” trials because it has been suggested that “same” matching invokes more holistic processing whereas “different” matching relies on analytical processing (Taylor, 1976). Hence, the “same” pairs provide an upper bound on degree of holistic processing for a given experimental condition.

This analogy with serial and parallel processing is useful because the idea of a serial comparison process suggests that the individual features are not processed independently. If faces are indeed processed holistically, as suggested by an abundance of evidence, then processing individual face components will be difficult and will likely result in a serial response function as illustrated by the green line in**Figure 1**. If, however, features can be processed independently from each other in an analytical fashion, then the response functions will shift toward a function with slope of 0 (red line in **Figure 1**). Of course, similarity functions may emerge that are neither purely parallel nor purely serial exhaustive (e.g., blue line).

A second condition was also tested – 2^nd^ order configural matching. Given the robust debate about whether inversion affects featural or 2^nd^ order processing differently (Rossion, 2009) or in the same manner (Riesenhuber and Wolff, 2009), a comparison of the two different kinds of perceptual information in the present framework can speak to this controversy. In addition, other tests of holistic processing of faces such as the composite effect (Young et al., 1987) and part-whole effect (Davidoff and Donnelly, 1990; Tanaka and Farah, 1993) result in both featural and 2^nd^ order changes in faces, making it difficult to assess the independent contributions of these changes to holistic face processing. As shown in **Figure 1B** the 2^nd^ order face (or house) pairs all had the same features, but the pairs differed in the spacing of the features in a systematic manner (see methods and detail shown in the figure). Again, if the face is perceived as an integrated percept, the spacing relations cannot be easily accessed as individual elements which will result in a serial comparison of the two faces in a pair. But if the individual spacing relations can be perceived independently from the rest of the face, then the comparison process will show evidence for parallel processing.

The central thesis of the present study is that adults will show the most evidence for processing faces as an integrated percept whereas younger children will show the most evidence for processing faces analytically or in a piecemeal fashion. With an integrated percept, access to and comparison of individual features across two faces, whether they are components like eyes, nose, etc., or spacing features, will be more difficult and result in relatively more serial processing, or sloped RT similarity functions. In contrast, if the percept of a face is not highly integrated, decomposing the face into constituent features will be less difficult and features may be processed in parallel, as indicated by flat RT similarity functions. In the present study, the focus is on the relative change in slopes of these similarity functions across different categories (faces versus houses), orientations (upright versus inverted), processing types (featural and 2^nd^ order) and age groups (adults, older children, younger children).

Given this conceptual framework the following hypotheses are tested:

(1) If adults and older children represent faces holistically, they should exhibit a stronger face inversion effect (collapsed over similarity) than younger children.
(2) (a) If adults and older children represent faces holistically, they should engage a serial comparison process as a function of similarity of the face pairs. Younger children should show more evidence for parallel processing, driven by more analytical processing. (b) When serial processing is present for upright faces (indicating holistic representations), houses and inverted faces will induce a bias toward parallel processing or weakening of serial processing.
(3) If inversion affects only 2^nd^ order configural face processing, then inverted faces will show a bias toward parallel processing only in this condition and not in the featural condition.

## Materials and Methods

### Participants

For Dataset 1, 18 healthy adults (mean age = 23.6 years, nine males), nine older children (mean age = 10.6 years, six males) and 10 younger children (mean age = 6.9 years, five males) participated in the featural condition. 38 healthy adults (mean age = 19.2 years, 18 males), 12 older children (mean age = 10.6 years, seven males) and 13 younger children (mean age = 7.2 years, five males) participated in the 2^nd^ order condition.

Dataset 2 was collected as part of a functional magnetic resonance imaging (fMRI) study. Fourteen healthy adults (mean age = 22.4 years, six males), 10 older children (mean age = 10.2 years, five males) and 23 younger children (mean age = 7.3 years, 12 males) participated in the featural condition. Eighteen healthy adults (mean age = 20.5 years, 10 males), seven older children (mean age = 10.7 years, four males) and five younger children (mean age = 6.7 years, two males) participated in the 2^nd^ order condition. The behavioral data for adults from this fMRI study has been published (Collins et al., 2012) but the analyses used in the present study were different from the published study.

All subjects had normal or corrected-to-normal visual acuity and normal color vision. In Dataset 1, 22% of the child participants were left-handed and 20% of the adults. For Dataset 2 all subjects were right-handed (as required for the fMRI study). Children completed the Peabody Picture Vocabulary Test (PPVT; Dunn and Dunn, 2007) and Expressive Vocabulary Test (EVT; Williams, 1997) and all children scored in the normal range. No participants reported neurological or psychiatric diagnoses, learning disability, medical conditions, or pregnancy.

Children provided assent and a parent provided informed consent before participating. Children and adults were compensated for participation but some adults received course credit instead of compensation. All procedures were approved by the University of Kentucky’s and Medical University of South Carolina’s Institutional Review Boards.

### Design and Stimuli

For Dataset 1, the 2 × 2 × 4 × 2 × 3 design had five independent variables: category (face, house), orientation (upright, inverted), and similarity (four levels of graded similarity, as illustrated in **Figures 1** and **2**) manipulated within subjects and processing type (featural, 2^nd^ order) and age group (younger children, older children, and adults) manipulated between subjects.

For Dataset 2, the 2 × 4 × 2 × 3 design had four independent variables: category and similarity manipulated within subjects and processing type and age group manipulated between subjects. Although Dataset 2 did not manipulate orientation, the data were used in a supplementary analysis to increase sample size and assess the reliability of the effects obtained with only Dataset 1.

Photo-realistic faces were constructed using FACES 4.0 software (IQ Biometrix, Redwood Shores, CA, USA) and house stimuli were created using Chief Architect 10.06a (Coeur d’Alene, ID, USA).

#### Featural Changes

Twenty-four faces or houses were initially constructed so that none of the features overlapped across these 24 stimuli. These were used as the basis for making featural changes and constructing stimulus pairs that varied in similarity.

For each original face, distracter faces were constructed so that 1, 2, 3, or 4 features (eyes, nose, mouth, or eyebrows) were replaced, yielding four similarity (sim) levels (and 96 unique faces: 24 original faces × 4 variants). Sim0–sim3 faces respectively shared 0–3 common features with the target face. The feature changed for each sim level was counterbalanced across all stimulus pairs so that feature replacement was not confounded with sim level. The same procedures were used for house features (door, steps, lower-level and upper-level windows). Forty-eight “same” pairs of faces or houses included two identical faces or houses, which were randomly selected from the pool of 96 face or house stimuli.

#### 2^nd^ Order Configural Changes

Twenty-four faces or houses were initially constructed so that none of the features overlapped across these 24 stimuli. The 2^nd^ order face changes were: (a) horizontal distance between the centroid of both eyes/brows (these features were moved together so that the brows were always aligned with the eyes), (b) vertical distance between centroid of nose and top of forehead, (c) vertical distance between centroid of mouth and top of forehead, and (d) vertical distance between center of two brows and top of forehead. For faces, an initial spacing of 2 SD from the norms published by (Farkas, 1994) was used, but was changed to a 3 SD spacing after 2 SD was identified as being too difficult to detect. The house changes were: (a) horizontal distance between the centroid of both lower windows, (b) horizontal distance between the centroid of both upper windows, (c) vertical distance between center of lower windows and bottom of roof, and (d) vertical distance between center of upper windows and bottom of roof. Again, the relation changed for each sim level was counterbalanced across all pairs to avoid confounding with sim level.

### Procedure

For Dataset 1 each participant completed 256 trials that required a response of “no” (e.g., different trials that varied across four similarity levels, sim0–sim3 trials in **Figures 1** and **2**) and 64 trials that required a response of “yes” (e.g., same trials that consisted of identical stimulus pairs, as in **Figure 1**). The 256 “no” trials consisted of 80 upright face pairs, 80 upright house pairs, 48 inverted face pairs, and 48 inverted house pairs. The 64 “yes” trials consisted of 20 upright face pairs, 20 upright house pairs, 12 inverted face pairs and 12 inverted house pairs. Some of the pairs used for upright trials (38 different pairs and 10 same pairs) were also used for inverted trials, with the remaining inverted trials consisting of unique stimulus pairs that were not used on upright trials. Each subject received a random order of the 320 trials, which were broken up into four blocks of 80 trials providing rest periods for the participants.

On each trial, participants saw either two faces or two houses for 2900 ms followed by a fixation interval for 520 ms. Participants indicated whether the two stimuli were the same (index finger) or different (middle finger) using a serial response box. Participants could respond at any point during the trial. The duration and trial length were fixed because we conducted the behavioral and fMRI study in parallel and wanted to equate the designs of the two studies (and fMRI studies necessarily require a fixed interval for responding). We also wanted a brief period in between trials to present a blank screen; otherwise the stimuli would appear in a consecutive stream which would greatly increase the difficulty of the task. No feedback was given about performance because the major goal was to study perception of faces rather than learning.

For Dataset 2 each participant completed 256 trials broken up into four runs of 64 trials each. Within each run, the 64 trials were broken up into eight blocks (4 similarity levels × 2 repetitions) of eight trials (5 “no” and 3 “yes” trials). Hence, of the 256 trials, 96 trials required a “yes” response and 160 required a “no” response. Two of the runs were face matching and two runs were house matching. The order of the four runs was counterbalanced across subjects. Participants had rest breaks between blocks and between runs.

### Analysis of Reaction Time and Error Rate

Reaction time on each correct trial was log_10_ transformed (logRT) to meet the assumption of normality for multivariate tests. Outliers were determined separately for each age group and processing type and defined by logRTs that were more than 3 SD above or below the mean. Outliers accounted for 0.06% of the data in adults and 1.7% of the data in children. Errors were defined as incorrect responses or response omissions and the average error rate per condition was used in analyses.

Analyzing logRT across age groups (for Hypotheses 1 and 2) as a function of similarity needs to address the concern of interpreting scale-dependent interactions (Salthouse and Hedden, 2002). Specifically, differences in logRT as a function of age group or experimental condition cannot be interpreted unless those differences occur at the same parts of the RT scale. Given that children and adults (usually) perform at different parts of the RT scale, we addressed this in each analysis in the following ways.

First, in the analyses for Hypotheses 1 and 3, each age group and processing type was analyzed separately so concerns about age differences in RT did not need to be accounted for directly in the analyses. Second, in the analysis for Hypotheses 2a and 2b, which compared age groups directly, an ANCOVA approach was used in which logRT or errors in the sim0 condition served as the covariate, similarity (sim1–sim3) was the repeated factor and age group was the between-subjects factor. Sim0 is the best candidate for a covariate because it represents a baseline level of performance in which all features or relations are different between the stimuli, but the RT would still reflect other cognitive operations (such as orientation to the stimuli, response selection and response execution) that may differ across age groups. “Same” trials were analyzed in separate ANCOVAs from “different” trials: sim0 was the covariate and age group was the between-subjects factor. Sim0 served as the covariate for “same” conditions in order to control for the cognitive operations that were not specific to faces or houses.

The design used in this study (Dataset 1) was a full factorial design with three within-subjects variables (category, orientation and similarity) and two between-subjects variables (age, processing type). However, we did not conduct a full factorial ANOVA for two reasons. First, there were not enough degrees of freedom to estimate the four-way and five-way interactions given the number of subjects in each age group (at least for the featural condition). Second, the ANCOVA approach used sim0 as the covariate for a given condition (such as upright faces or inverted houses). With the full factorial design it would not be clear how to specify a single covariate for all of the experimental conditions or how to map the sim0 condition to different Category × Orientation combinations. Therefore, each hypothesis was tested with analyses for some subset of the variables (described for each hypothesis below). When interactions with similarity were present, simple effects analysis (Keppel and Zedeck, 1989) of similarity was conducted. The simple effects analysis would indicate whether the similarity function was significant for a given condition. Polynomial contrasts were then conducted to indicate whether the similarity function followed a linear trend.

Although error rates are not necessarily subject to the same concern of scale-dependent interactions (but see Salthouse and Hedden, 2002), we used the same ANCOVA approach for the analysis of error rates to keep the analyses consistent. However, we used the RT measure in order to examine serial versus parallel processing as that is the most typical measure used to estimate these processes.

## Results

### Hypothesis 1: Adults and Older Children should Show Stronger Face Inversion Effects than Younger Children

Following other findings in the literature, adults and older children were expected to show a stronger face inversion effect than younger children. This analysis only used data from Dataset 1 as that was the only dataset with an inversion manipulation. In addition, featural and 2^nd^ order conditions were analyzed separately given that initial inspection of error rates revealed that 2^nd^ order matching was more difficult.

#### Featural Processing

**FIGURE 3:**
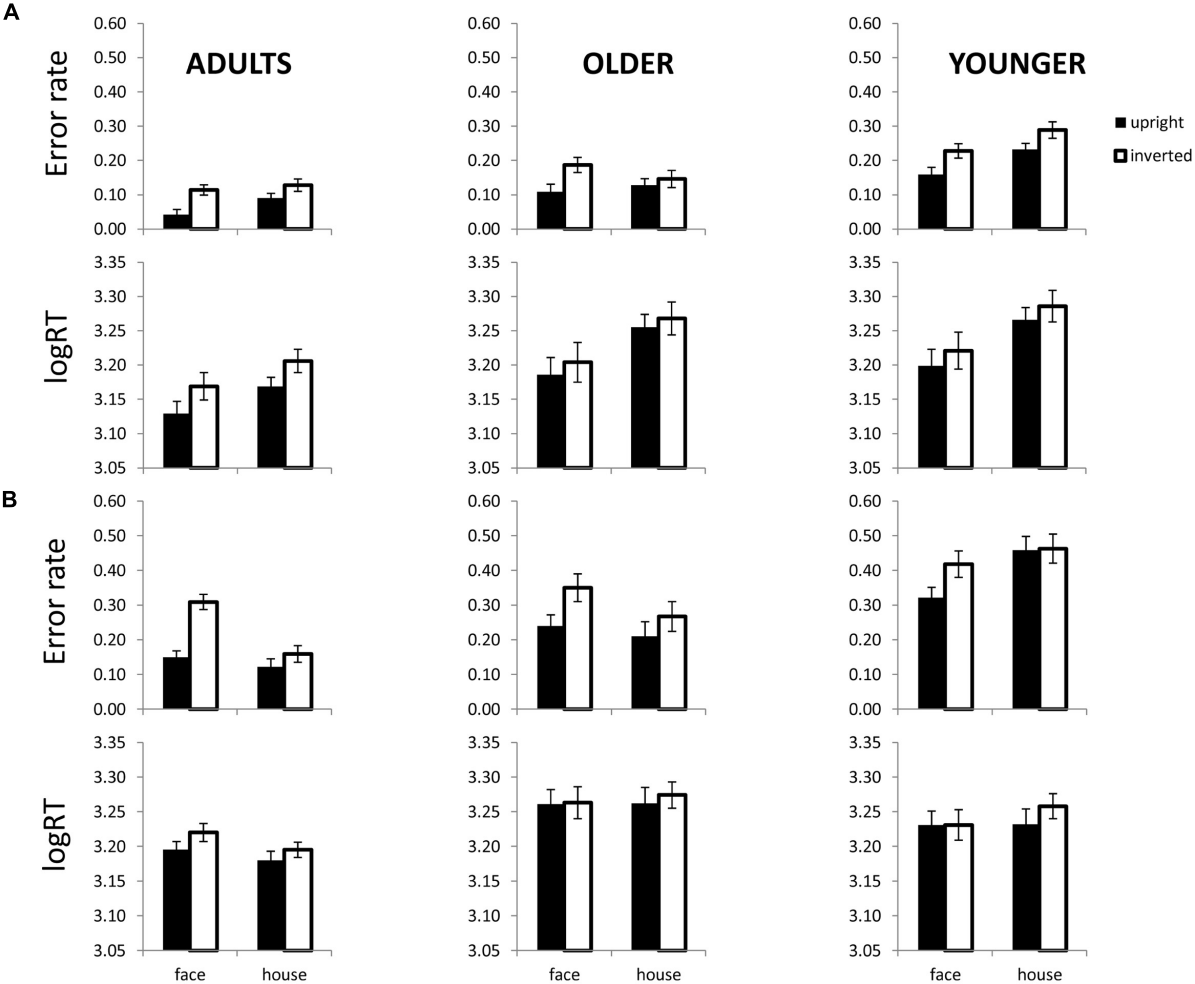
**Face and house matching performance as a function of inversion in each age group and for each processing type. (A)** Shows error rates (top) and logRT (bottom) for each age group in the featural condition. **(B)** Shows error rates (top) and logRT (bottom) for each age group in the 2^nd^ order condition. Error bars are SE of the mean.

Repeated measures ANOVAs with logRT and errors as dependent variables and category (face, house) and orientation (inverted, upright) were conducted separately for adults, older children and younger children. The presence of a Category × Orientation interaction served as the main test of the hypothesis. As shown in **Figure 3A** adults and older children showed a trend for a greater inversion effect for featural faces than for featural houses with respect to errors, but younger children did not show this interaction. The Category × Orientation interaction was marginal in adults, *F*(1,17) = 3.2, *p* = 0.089, and older children, *F*(1,8) = 4.1, *p* = 0.076, but not significant for younger children for errors (*p* = 0.727). For logRT, the Category × Orientation interaction was not significant (*p*’s > 0.77).

#### 2^nd^ order processing

For 2^nd^ order configural faces and houses, all three age groups showed a trend for a face inversion effect with respect to errors (**Figure 3B**). In adults, the Category × Orientation interaction was significant for errors, *F*(1,37) = 53.8, *p* = 0.0001. The interaction was marginal in both older children, *F*(1,11) = 3.9, *p* = 0.073, and younger children, *F*(1,12) = 3.6, *p* = 0.082 for errors. The interaction was not significant for any age group for logRT (*p*’s > 0.27).

### Analysis of Similarity for Errors

#### Featural Processing

**FIGURE 4:**
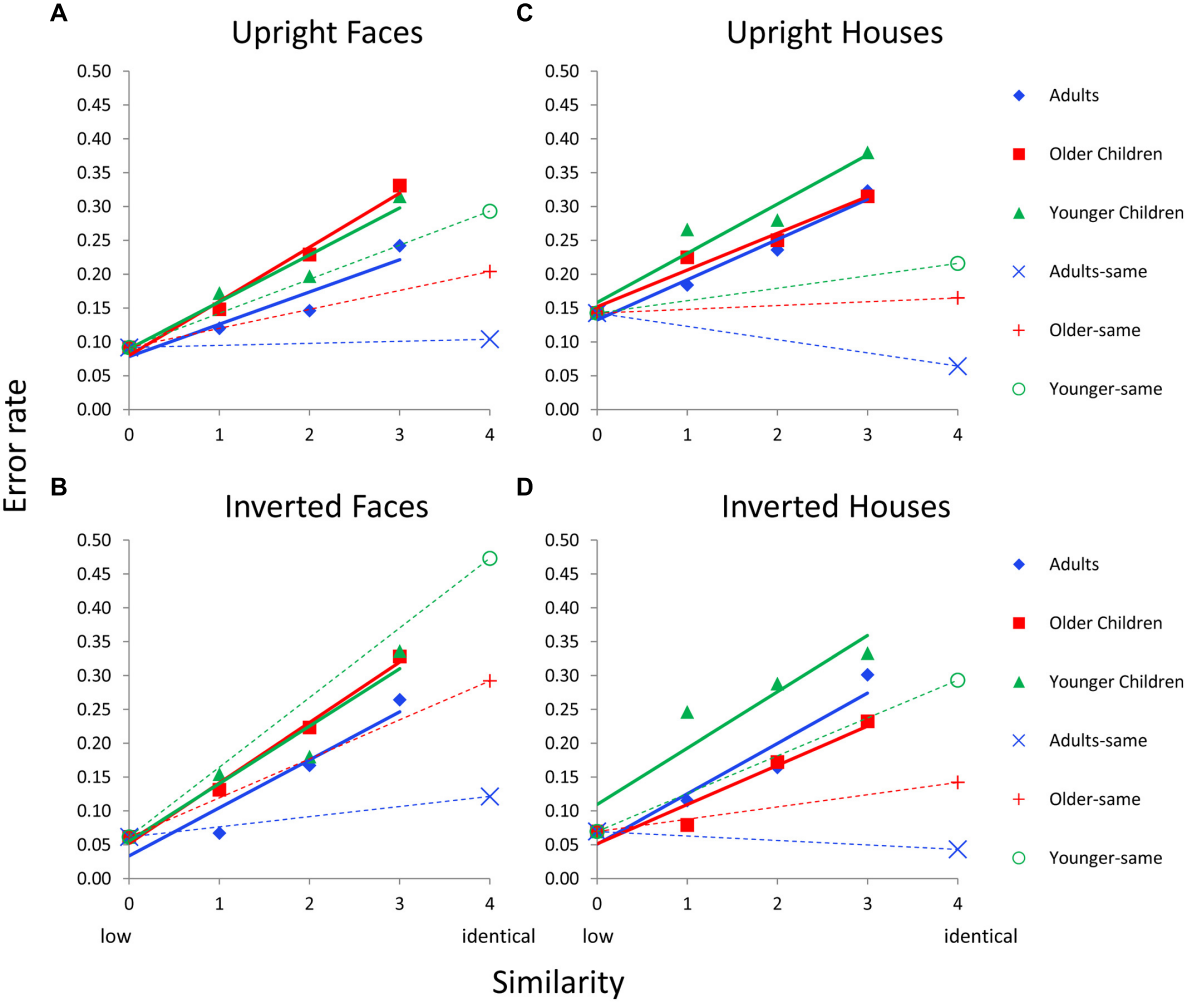
**Face and house matching error rates as a function of inversion, similarity, and age group for the featural condition. (A)** Results for upright faces. **(B)** Results for inverted faces. **(C)** Results for upright houses. **(D)** Results for inverted houses. In each panel, similarity functions for “different” trials are indicated as solid lines and filled symbols and similarity functions for “same” trials are indicated with dotted lines and hollow symbols. In a given panel, each age group’s similarity function is scaled to the same adjusted mean value in the sim0 condition. Error bars are not shown given the complexity of the graphs.

**Figure 4** shows errors as a function of similarity for each age group and each Category × Orientation (i.e., upright faces, inverted faces, upright houses, inverted houses) condition for featural matching for Dataset 1 (which manipulated orientation). Each age group’s error function is adjusted based on sim0 error (the covariate) so this value is the same for all age groups and conditions on a given graph. Solid colored lines indicate error functions for “different” trials; dotted colored lines indicate error functions for “same” trials. The primary goal of this analysis was to determine whether there were age differences in the slopes of the similarity functions; hence, the Age × Similarity interaction was of primary interest for “different” trials and the main effect of age was of interest for “same” trials.

For “different” trials, an Age × Similarity ANCOVA was conducted with sim0 as the covariate separately for each Category × Orientation combination. The Age × Similarity interaction was not significant for any condition for “different” trials (*p*’s > 0.48). For same trials, an ANCOVA was conducted with age as the independent variable and sim0 as the covariate. The main effect of age was marginal for upright faces, *F*(2,37) = 3.1, *p* = 0.062, significant for inverted faces, *F*(2,37) = 6.2, *p* = 0.005, upright houses, *F*(2,37) = 3.9, *p* = 0.032, and inverted houses, *F*(2,37) = 5.4, *p* = 0.009.

#### 2^nd^ order Processing

**FIGURE 5:**
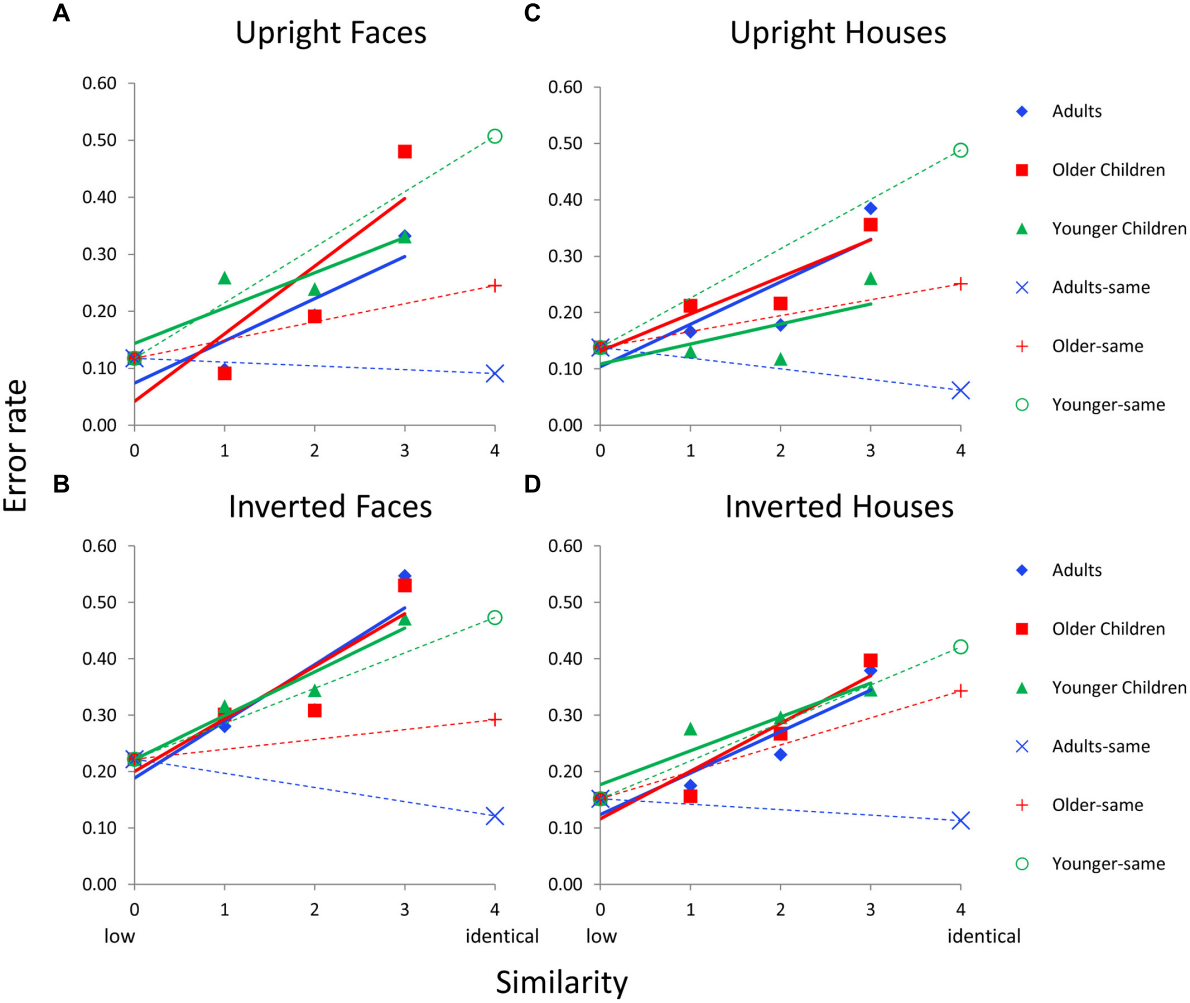
**Face and house matching error rates as a function of inversion, similarity, and age group for the 2^nd^ order condition. (A)** Results for upright faces. **(B)** Results for inverted faces. **(C)** Results for upright houses. **(D)** Results for inverted houses. In each panel, similarity functions for “different” trials are indicated as solid lines and filled symbols and similarity functions for “same” trials are indicated with dotted lines and hollow symbols. In a given panel, each age group’s similarity function is scaled to the same adjusted mean value in the sim0 condition. Error bars are not shown given the complexity of the graphs.

The same ANCOVAs conducted for featural processing in Section “Featural Processing” were conducted for 2^nd^ order processing. For The Age × Similarity interaction was only significant for upright faces, *F*(4,118) = 6.0, *p* = 0.0001, on “different” trials (**Figure 5**). On “same” trials, the main effect of age was significant for upright faces, *F*(2,63) = 24.7, *p* = 0.0001, inverted faces, *F*(2,63) = 3.3, *p* = 0.045, upright houses, *F*(2,63) = 21.0, *p* = 0.0002, and inverted houses, *F*(2,63) = 11.2, *p* = 0001.

In summary, although there were no specific hypotheses with respect to error rates, this analysis was presented to show that adults perform the task more accurately than children, as expected. However, there were few age differences in similarity functions for either featural or 2^nd^ order faces. Only upright featural houses and upright 2^nd^ order faces showed interactions with age. Age effects were much more pronounced on “same” trials, with adults showing lower error rates. One important point from this analysis was that, even though error rates were quite high for some conditions, the primary analysis used RT only on *correct* trials. Therefore, in subsequent analyses, speed-accuracy tradeoffs are not driving the effects.

### Hypothesis 2a: Adults and Older Children will Show More Evidence for Serial Processing than Younger Children

This analysis was conducted to test Hypothesis 2a, which predicts that adults and older children should engage a serial comparison process as a function of similarity of the face pairs (and show more sloped similarity functions with a positive linear trend) whereas younger children should show more evidence for parallel processing (and show flatter similarity functions and no positive linear trend). To test this hypothesis, an Age × Similarity ANCOVA was conducted with sim0 as the covariate separately for each Category × Orientation combination. The presence of a Similarity × Age interaction was the primary test of the hypothesis. When this interaction was significant, simple main effects (Keppel and Zedeck, 1989) of similarity for each age group were also examined to determine whether the similarity function was positive and linear as an indication of serial processing. The linear trend was assessed using planned polynomial contrasts. Results are presented first for Dataset 1, which manipulated orientation in addition to similarity and category.

#### Featural Processing

**FIGURE 6:**
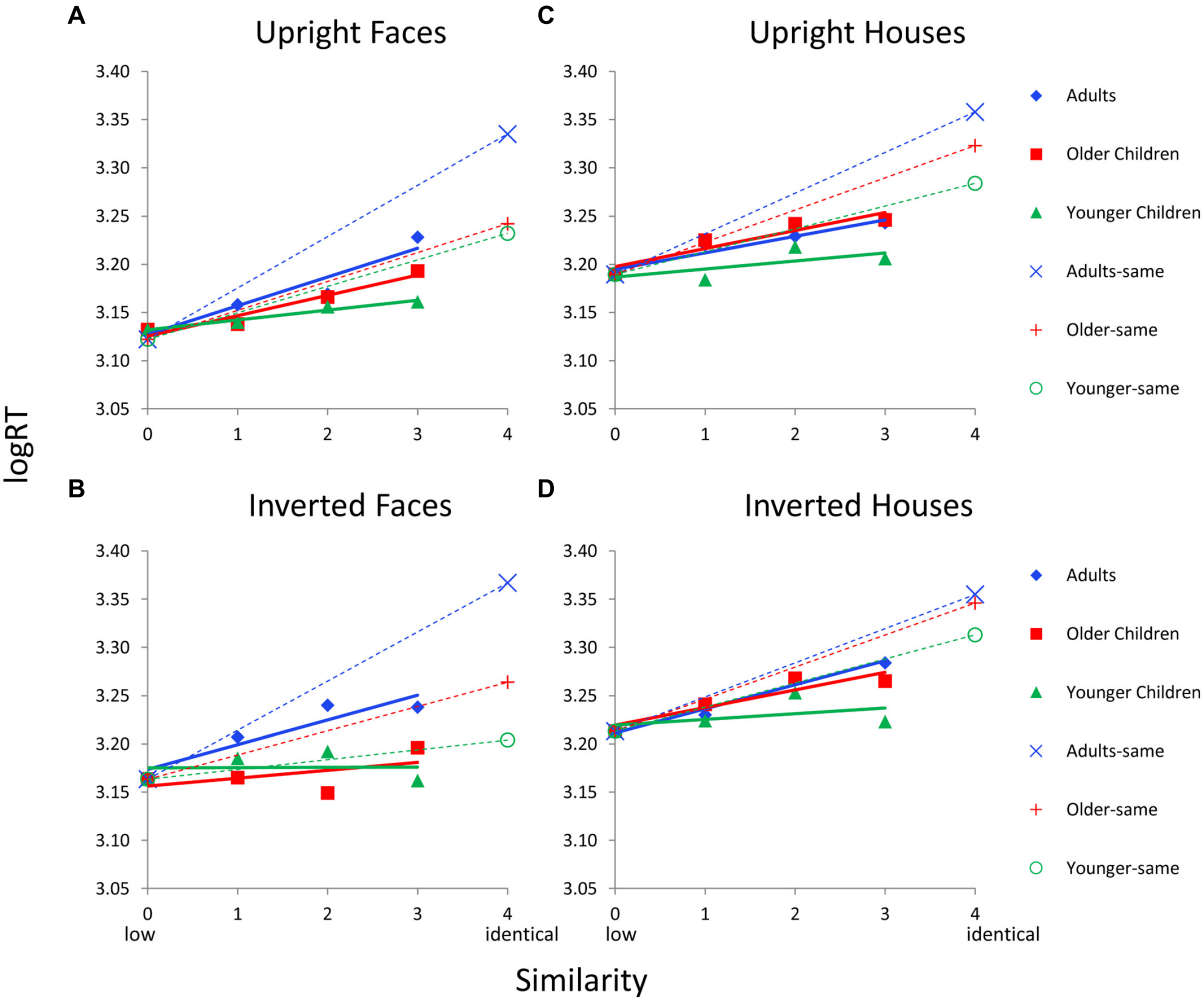
**Face and house matching logRT as a function of inversion, similarity, and age group for the featural condition. (A)** Results for upright faces. **(B)** Results for inverted faces. **(C)** Results for upright houses. **(D)** Results for inverted houses. In each panel, similarity functions for “different” trials are indicated as solid lines and filled symbols and similarity functions for “same” trials are indicated with dotted lines and hollow symbols. In a given panel, each age group’s similarity function is scaled to the same adjusted mean value in the sim0 condition. Error bars are not shown given the complexity of the graphs.

**Figure 6** shows logRT as a function of similarity by age group and by each Category × Orientation condition for featural faces. Each age group’s RT function is adjusted based on sim0 logRT (the covariate) so this value is the same for all age groups on a given graph. Solid colored lines indicate RT functions for “different” trials; dotted colored lines indicate RT functions for “same” trials. Interestingly, across all Category × Orientation conditions, younger children show evidence for parallel processing, with similarity functions that are nearly flat. Parallel processing seems to persist across inversion and category manipulations. In contrast, similarity functions for adults have steeper slopes than those for children, especially for face stimuli. Older children show a pattern that is intermediate to adults and younger children for upright faces, but that is similar to younger children for inverted faces. Older children look similar to adults for house stimuli.

The ANCOVAs, however, revealed age group differences in RT functions only for face stimuli for “different” trials. For upright faces, the Similarity × Age Group interaction was significant, *F*(4,66) = 2.7, *p* = 0.044. However, the simple effect of similarity was not significant for any age group. For inverted faces, the Similarity × Age Group interaction was significant, *F*(4,66) = 2.5, *p* = 0.049, and the simple effect of similarity was significant only for younger children (*p* = 0.047) and the trend was marginally linear (*p* = 0.05). However, this effect was driven by sim3 having faster RTs than the other sim levels (**Figure 6B**) so the linear trend was in the negative direction, which is not consistent with serial processing. For house stimuli, the Similarity × Age interaction was not significant.

Age group differences seemed to be even more pronounced on “same” trials. Adults always showed the highest RT (but older children were similar to adults for inverted houses), a pattern suggesting a trend toward serial exhaustive search. Children show a trend toward serial processing for “same” trials in most conditions as indicated by a longer RT for same responses than for the highest similarity level on different trials, except for inverted faces, where younger children show a tendency toward parallel processing (i.e., RT for same is not longer than RT for different trials). For “same” trials, the main effect of age was significant for upright faces, *F*(2,37) = 8.3, *p* = 0.001, inverted faces, *F*(2,37) = 18.1, *p* = 0.0001, upright houses, *F*(2, 37) = 5.0, *p* = 0.013, but not inverted houses.

Data from Dataset 2 were combined with the data from Dataset 1 and analyses were rerun. As mentioned, these analyses only applied to upright stimuli as Dataset 2 did not manipulate orientation. The ANCOVAs revealed age group differences in RT functions only for featural face stimuli for “different” trials: the Similarity × Age Group interaction was marginal, *F*(4,160) = 2.0, *p* = 0.095, but the simple effect of similarity was significant for adults, *p* < 0.009 (linear trend, *p* = 0.003). For “same” trials, the main effect of age was significant for upright faces, *F*(2,84) = 12.4, *p* = 0.0001, and upright houses, *F*(2,86) = 8.8, *p* = 0.0001.

#### 2^nd^ Order Processing

**FIGURE 7:**
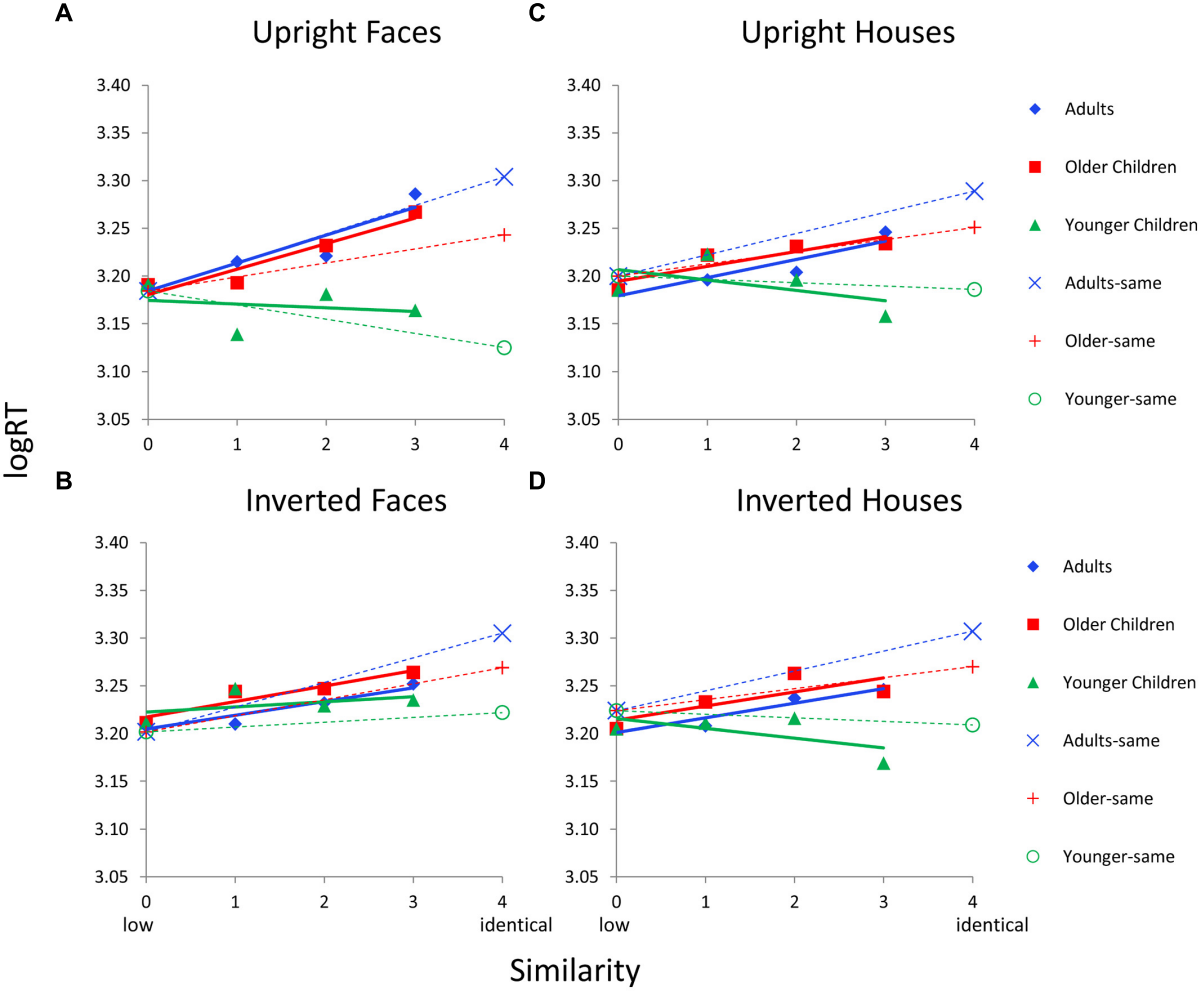
**Face and house matching logRT as a function of inversion, similarity, and age group for the 2^nd^ order condition. (A)** Results for upright faces. **(B)** Results for inverted faces. **(C)** Results for upright houses. **(D)** Results for inverted houses. In each panel, similarity functions for “different” trials are indicated as solid lines and filled symbols and similarity functions for “same” trials are indicated with dotted lines and hollow symbols. In a given panel, each age group’s similarity function is scaled to the same adjusted mean value in the sim0 condition. Error bars are not shown given the complexity of the graphs.

**Figure 7** shows logRT as a function of similarity by age group and by each Category × Orientation condition for 2^nd^ order configural stimuli. Younger children again show flatter similarity functions, or even negative-going patterns for some conditions, compared to older children and adults. Older children show functions that have similar slopes to adults across all conditions. The ANCOVAs revealed age group differences in RT functions only for upright stimuli. For upright faces, the Similarity × Age Group interaction was significant, *F*(4,118) = 3.3, *p* = 0.017, and the simple effect of similarity was significant for adults (*p* < 0.031) and older children (*p* < 0.013) but the linear trend was only significant in adults (*p* = 0.016). For upright houses, the Similarity × Age interaction was significant, *F*(4,118) = 9.0, *p* = 0.0001, but the simple effect of similarity was only marginally significant for older children (*p* = 0.053, no linear trend) and not significant for adults or younger children.

For “same” trials, the main effect of age was significant for upright faces, *F*(2,63) = 9.8, *p* = 0.0001, inverted faces, *F*(2,62) = 4.6, *p* = 0.014, upright houses, *F*(2,63) = 6.7, *p* = 0.002, and inverted houses, *F*(2,63) = 4.3, *p* = 0.018. Similar to the finding for featural faces, adults always have a longer RT on same trials than on different trials and younger children have an RT on same trials that is comparable to or faster than different trials.

Data from Dataset 2 were combined with the data from Dataset 1 and analyses were rerun. For 2^nd^ order upright faces, the Similarity × Age Group interaction was significant, *F*(4,178) = 4.0, *p* = 0.004, and the simple main effect of similarity was significant for adults (*p* < 0.006) and marginal for older children (*p* = 0.086), but the linear trend was only significant in adults (*p* = 0.005). For 2^nd^ order upright houses, the Similarity × Age Group interaction was significant, *F*(4,178) = 11.1, *p* = 0.0001, but the simple effect of similarity was not significant for any age group. For “same” trials, the main effect of age was significant for upright faces, *F*(2,84) = 12.4, *p* = 0.0001, and upright houses, *F*(2,93) = 9.6, *p* = 0.0001.

#### Analysis of Untransformed Reaction Time

The interpretation of positively sloped similarity functions as evidence for serial processing may be questioned if log-transformed RTs are used, as in the present study. In other words, a log transformation is a non-linear transformation, so the relation between similarity and RT cannot necessarily assumed to be linear, which is an important assumption for serial processing. To address this, we conducted the analyses for Hypothesis 2a using the raw, untransformed RT (only for correct responses and with outliers removed, as was the case for log-transformed RTs) and the results are fundamentally the same (see Supplement). Importantly, the log-transformed and untransformed RT values yield a similar pattern of similarity functions with respect to age group. Because the log-transformed RTs lead to the same conclusions we would have reached using untransformed RTs, the remaining analyses were conducted using log-transformed RTs.

### Hypothesis 2b: Serial Processing will be Weaker for Houses and Inverted Faces

Hypothesis 2b states that when serial processing is present for upright faces (indicating holistic representations), houses and inverted faces will induce a bias toward parallel processing or weaker serial processing. Serial processing was only evident for 2^nd^ order upright faces (according to the analyses for Hypothesis 2a in Section “Hypothesis 2a: Adults and Older Children will Show More Evidence for Serial Processing than Younger Children”). However, that analysis compared similarity functions across age but did not directly compare categories or orientations. The analysis for Hypothesis 2b requires comparing similarity functions across categories or across orientation conditions. These analyses were thus conducted within each age group that showed some evidence for serial processing of upright faces; namely, adults and older children (but the effect in older children was marginal and the linear trend did not reach significance). Also, because different age groups were not compared with each other in this analysis, sim0 was not a covariate but instead was included as a level of the independent variable of similarity.

The Similarity (sim0-3) × Category (upright face, upright house) ANOVA revealed a significant interaction only in adults, *F*(3,111) = 4.0, *p* = 0.01, with houses invoking a bias toward parallel processing (**Figure 7**). Although a similar pattern is apparent in older children, this interaction was not significant. The Similarity × Orientation (upright face, inverted face) ANOVA also revealed a significant interaction only in adults, *F*(3,111) = 8.1, *p* = 0.0001, with inverted faces invoking a bias toward parallel processing. Again, older children showed the same pattern but the interaction was not significant. Hence, serial processing is only significant in adults for 2^nd^ order upright faces. House and inverted face stimuli induce more parallel processing in adults (or weaken serial processing). Older children show a similar pattern as adults, but the effects do not reach significance. Younger children show no evidence for serial processing for any of the stimuli or conditions.

### Hypothesis 3: Inversion May Affect 2^nd^ Order Processing More than Featural Processing

Hypothesis 3 states that inversion may affect 2^nd^ order processing more than featural processing. Given that 2^nd^ order upright faces were the only stimulus that invoked serial processing (in adults) and serial processing was weaker with inversion (the significant Similarity × Orientation interaction in adults), this hypothesis would be supported based on analyses above. However, a Similarity (sim0–sim3) × Orientation (upright, inverted) × Processing Type (featural, 2^nd^ order) ANOVA was conducted separately in adults to directly compare similarity functions for different processing types and orientations. Although 2^nd^ order faces appear to be more difficult, the similarity functions overlap on the RT scale. Therefore, a covariate was not used in this analysis. The Similarity × Orientation × Processing Type interaction was significant, *F*(3,162) = 3.6, *p* = 0.022. As shown in **Figures 6** and **7**, the similarity functions for featural faces have similar slopes for upright versus inverted faces, but the similarity functions for 2^nd^ order faces are different, with serial processing being weaker with inversion.

## Discussion

The present study examined perceptual matching performance in children and adults to further characterize developmental changes in processing facial information. The experiments manipulated many important factors that have been examined in prior studies of face development, including featural versus 2^nd^ order processing, inversion and category, but the novel contribution was considering how these factors impact serial versus parallel processing which is a marker of the degree to which holistic processing is engaged. In general, several prior findings were replicated but new insights into the development of face processing also emerged. Each hypothesis is discussed in turn.

### Findings for Hypothesis 1: Adults Show Stronger Face Inversion Effects than Younger Children

If adults and older children represent faces holistically, they should exhibit stronger face inversion effects (collapsed over similarity) than younger children. This hypothesis was somewhat supported. Face inversion effects were observed only with respect to errors and not logRT. For featural stimuli, adults and older children showed marginally significant face inversion effects but younger children did not. For 2^nd^ order configural stimuli, adults showed a significant face inversion effect and older and younger children showed marginally significant face inversion effects. In summary, adults were the only group to show a statistically significant face inversion effect, and only in the 2^nd^ order condition. The marginal inversion effects in children are not surprising given the many findings that younger children show lessened face inversion effects. In addition, inversion effects appeared to be more pronounced for 2^nd^ order faces as reported by many others (see Rossion, 2009 for review). However, 2^nd^ order faces were indeed more difficult to differentiate, as the upright conditions for featural and 2^nd^ order faces did not appear to be equated. Given this, we suggest that the question of whether inversion differentially affects featural and 2^nd^ order processing be answered in the context of similarity functions and parallel versus serial processing (see Hypothesis 3).

### Findings for Hypothesis 2a: Adults and Older Children Show More Evidence for Serial Processing than Younger Children

If adults and older children represent faces holistically, they should engage a serial comparison process as a function of similarity of the face pairs. Younger children should show more evidence for parallel processing, driven by more analytical processing. This hypothesis was largely confirmed. Similarity functions for younger children showed evidence for parallel processing whereas similarity functions for adults and older children showed evidence for serial processing, most strongly in the 2^nd^ order condition. Similarity functions were different in adults and children for face stimuli in the featural condition and for upright stimuli in the 2^nd^ order condition. Older children showed similar patterns as adults and significant simple effects of similarity for upright 2^nd^ order faces, but the linear trend was not significant. In fact, the similarity effect was linearly increasing (indicating serial processing) only for “adults” 2^nd^ order and featural upright face conditions.

These findings suggest that older children show more adult-like processing of 2^nd^ order information than featural information, with a holistic representation of faces that is more strongly linked to 2^nd^ order information. Featural information is not as strongly integrated into a holistic representation in older children because they invoked a more “immature” strategy of parallel processing for featural faces. It seems, then, that (a) younger children show the weakest evidence for holistic representations, (b) older children show some evidence for holistic representations, but those representations incorporate 2^nd^ order relations more than featural representations, and (c) adults show the strongest evidence for holistic representations that incorporate both 2^nd^ order relations and, to some extent, featural information.

In some sense, these findings appear to be at odds with the conclusion from many studies that featural processing of faces develops sooner than 2^nd^ order processing (see Mondloch et al., 2010 for review). We suggest that this apparent discrepancy likely reflects a transitional phase in older children in which the holistic representation includes both featural and 2^nd^ order information but the degree to which that information is integrated is weaker compared to adults. Therefore, featural information is somewhat more accessible for analytical processing in older children, but at the same time, the 2^nd^ order information is less accessible.

### Findings for Hypothesis 2b: Serial Processing was Weaker for Houses and Inverted Faces for Adults

When serial processing is present for upright faces (indicating holistic representations), houses and inverted faces will induce a bias toward parallel processing or weakening of serial processing. This hypothesis was confirmed only for adults. Older children showed patterns consistent with adults, but these patterns were not statistically significant. Younger children process upright faces in an analytical manner so neither inversion nor house stimuli could induce more parallel processing. These findings are indeed in line with some of the earliest studies showing that piecemeal or analytical processing of faces is predominant in young children and less so in adults (Carey and Diamond, 1977; Schwarzer, 2000). To our knowledge, however, this has not been demonstrated using a serial versus parallel processing framework. This finding is also consistent with attenuated face inversion effects in younger children both in the present study and the literature (Carey and Diamond, 1994; Schwarzer, 2000; Brace et al., 2001; Joseph et al., 2006; Meinhardt-Injac et al., 2014). Younger children process faces in a similar analytical manner as non-faces (Schwarzer, 2002); therefore, inversion has less effect on performance because inversion does not disrupt piecemeal processing.

### Findings for Hypothesis 3: Inversion Affects 2^nd^ Order Processing More than Featural Processing

If inversion affects only 2^nd^ order configural face processing, then inverted faces will show a bias toward parallel processing only in this condition and not in the featural condition. This hypothesis was examined to address the debate as to whether inversion affects 2^nd^ order processing more than featural processing (Rossion, 2009) or whether inversion affects both kinds of processing equally (Riesenhuber and Wolff, 2009). The present findings are more consistent with the suggestion by Rossion (2009) that inversion affects 2^nd^ order processing more. This was evident in the different slopes for similarity functions for 2^nd^ order faces, but parallel slopes for featural faces, as a function of inversion. However, the finding that inversion induces a change in the intercept for featural faces (**Figure 6**) while preserving the slope is consistent with suggestions that inversion does not invoke qualitatively different processing (Riesenhuber et al., 2004; Sekuler et al., 2004), at least for featural faces. On the other hand, for 2^nd^ order faces, inversion does not change the intercept but does change (i.e., weaken) the slope of the similarity function, indicating a shift away from serial to parallel processing. As noted by both sides of this debate, many of the findings depend on a range of different factors from defining what constitutes “features” or “face components” to different task demands. While the present findings do not resolve this debate, they do outline some conditions under which inversion induces a baseline shift in performance (featural information) versus inducing qualitatively different processing (2^nd^ order information).

### Limitations of the Present Study

One alternative explanation for the finding of flat similarity functions in younger children is that this represents a ceiling effect such that the task was so difficult that younger children needed to take a maximal amount of time to make correct perceptual decisions. However, if ceiling effects (and flat similarity functions) reflected difficulty with the task then children should show ceiling effects for conditions that adults also found very difficult. In particular, adults showed the slowest responding on “same” trials for any given condition and these responses were even slower than the difficult sim3 condition. In contrast, younger children show “same” responses that are on par with the sim3 condition or even faster than the sim3 condition for 2^nd^ order faces. This suggests that a different strategy is driving the similarity functions in younger children and adults, rather than a ceiling effect. Namely, because children are able to process features in parallel, they need not engage a serial exhaustive comparison process and can process the features simultaneously. Adults, in comparison, show evidence for a serial processing (and possibly serial exhaustive) strategy on “same” trials because RT is greater than or equal to RT in the sim3 condition.

Another potential limitation of the study was relatively small sample size, especially for the older children group. There is potentially greater heterogeneity in this age range (10–11 years) if perceptual processes engaged for faces are transitioning from a more immature pattern to a more adult-like pattern. Although we attempted to maximize sample size by including a second dataset, potential greater heterogeneity in this age would best be addressed with a larger sample. In this case, some of the adult-like patterns observed for older children may turn out to be significant.

## Summary and Conclusion

Using the conceptual framework of serial versus parallel processing as in other cognitive domains like selective attention and short-term memory scanning, the present study showed that holistic processing of faces matures during childhood. Younger children more often engaged parallel processing of individual face components and spacing relations than older children and adults. In contrast, adults more often engaged serial processing which is an index of holistic perception of faces. Older children showed a transitional pattern: their similarity functions often resembled that of adults, but effects did not always emerge as significant. We suggest that the findings in older children are driven by heterogeneity in performance across subjects precisely because they are in a transitional stage. Some older children exhibit adult-like holistic processing whereas other older children still exhibit a more immature analytical or piecemeal processing approach.

Holistic processing of upright faces in adults was reduced by inversion, primarily for 2^nd^ order faces. This finding maps onto the suggestion that inversion has a more pronounced effect on 2^nd^ order (spacing) information processing than on featural processing (Rossion, 2009). We suggest that this more pronounced effect is driven by a shift from holistic to more analytical processing with inversion. However, inversion induced a baseline shift in processing featural faces suggesting that the same process is engaged for upright and inverted featural processing (Riesenhuber and Wolff, 2009).

Development of face processing involves maturation of perceptual processes related to integrating featural and 2^nd^ order information into a unified, holistic representation. Younger children had weak holistic representations given that they engaged parallel processing of individual face features and relations in all experimental conditions. Older children most often resembled adults showing some evidence for holistic representations that integrated 2^nd^ order information. These findings map onto prior research findings but also point toward future and continued investigations of the circumstances that drive the use of 2^nd^ order and featural information for a given face task.

## Conflict of Interest Statement

The authors declare that the research was conducted in the absence of any commercial or financial relationships that could be construed as a potential conflict of interest.
