# Supplementary material for: Developmental changes in analytic and holistic processes in face perception
Source: Front Psychol. 2015 Aug 7;6:1165. doi: 10.3389/fpsyg.2015.01165 (PMC4528094; doi:10.3389/fpsyg.2015.01165)
Supplement: Supplementary file 1 [file Data_Sheet_1.PDF]

Supplemental Materials for

"Developmental changes in analytic and holistic processes in face perception"

To demonstrate that the similarity functions obtained using log-transformed RTs are fundamentally the same as the similarity functions obtained using untransformed RTs, this supplement reran all of the analyses for Hypothesis 2a using untransformed RTs.

1. Featural processing

Figure S1 shows RT as a function of similarity by age group and by each Category x Orientation condition for featural faces. Each age group's RT function is adjusted based on sim0 RT (the covariate) so this value is the same for all age groups on a given graph. Solid colored lines indicate RT functions for "different" trials; dotted colored lines indicate RT functions for "same" trials. Although it appears that adults, older children, or both, show more sloped similarity functions than younger children, the Similarity x Age interaction was not significant for any condition. In the analysis of log-transformed RT in the main paper, the Similarity x Age interaction was significant for some conditions. However, no age group showed a simple effect of similarity in which the slope was positive and linear. Therefore, the results using untransformed RTs are the same as the results with log-transformed RTs. As illustrated in Figure S1, the patterns of similarity functions by age are very similar to the patterns that emerged for log-transformed RTs.

For "same" trials, the main effect of age was significant for upright faces, $F(2, 37) = 4.7$, $p = .016$, inverted faces, $F(2, 37) = 14.0$, $p = .0001$, upright houses, $F(2, 37) = 3.6$, $p = .037$, but not inverted houses. Adults always had the longest RT and younger children always had the shortest RT. These results are identical to the results using log-transformed RT.

Data from Dataset 2 were combined with the data from Dataset 1 and analyses were rerun. The Similarity x Age interaction was not significant for either upright faces or upright houses. This result is somewhat different from the result using log-transformed RTs in that the Similarity x Age interaction was marginally significant for upright faces. However, the simple effect of similarity was significant for adults for upright faces, $p < .01$, and the linear trend was significant using untransformed RTs ($p = .006$), similar to the finding with log-transformed RTs.

For "same" trials in the combined dataset, the main effect of age was significant for upright faces, $F(2, 84) = 11.0$, $p = .0001$ and upright houses, $F(2, 86) = 7.2$, $p = .001$. Adults always have the longest RT and younger children always have the shortest RT. These results are identical to the results using log-transformed RT.

In summary, the results using untransformed RTs lead to the conclusion that only adults showed evidence for serial processing of upright faces for "different" trials when both datasets were combined, which is the same finding that emerged using log-transformed RTs. Also, adults showed more evidence for serial processing on "same" trials, similar to the findings using log-transformed RTs.

2. 2nd order processing

Figure S2 shows RT as a function of similarity by age group and by each Category x Orientation condition for 2nd order configural stimuli. Younger children show flatter similarity functions, or even negative-going patterns for some conditions, compared to older children and adults. Older children show functions that have similar slopes to adults across most conditions. The ANCOVAs revealed age group differences in RT functions for upright faces, upright houses, and inverted houses. For upright faces, the significant Similarity x Age Group interaction, $F(4, 118) = 3.8$, $p = .006$, revealed simple effects of similarity for adults ($p < .017$) and older children ($p < .002$) and the linear trend was significant in both adults ($p = .010$) and older children ($p = .035$). Results are nearly identical to those using log-transformed RTs with the exception that the linear trend was not significant for older children for upright faces. For upright houses, the significant Similarity x Age interaction, $F(4, 118) = 4.0$, $p = .004$, revealed a marginal simple effect of similarity only in adults ($p = .076$, linear trend: $p = .069$). In contrast, log-transformed RTs revealed a marginal simple effect of similarity in older children. For inverted houses, the significant Similarity x Age interaction, $F(4, 114) = 3.1$, $p = .018$, revealed a simple effect of similarity only in adults ($p = .037$, linear trend: $p = .046$). This result is different from the results using log-transformed RTs in that the Similarity x Age interaction was not significant.

For “same” trials, the main effect of age was significant for upright faces, $F(2, 63) = 10.4$, $p = .0001$, inverted faces, $F(2, 63) = 5.0$, $p = .009$, upright houses, $F(2, 63) = 3.4$, $p = .04$, and marginal for inverted houses, $F(2, 63) = 3.0$, $p = .058$. Adults always have a longer RT on same trials than on different trials and younger children have an RT on same trials that is comparable to or faster than different trials. These results are the same as those using log-transformed RTs.

Data from Dataset 2 were combined with the data from Dataset 1 and analyses were rerun. For 2nd order upright faces, the Similarity x Age Group interaction was significant, $F(4, 178) = 3.6$, $p = .007$, and the simple effect of similarity was significant for adults ($p < .005$) and older children ($p = .001$), but the linear trend was only significant in adults ($p = .004$). These results are nearly the same as the results using log-transformed RTs. For 2nd order upright houses, the Similarity x Age Group interaction was significant, $F(4, 176) = 4.7$, $p = .001$, and the simple effect of similarity was significant for adults ($p = .03$, linear trend: $p = .023$). Log-transformed RTs did not show a significant linear trend for any age group. For “same” trials, the main effect of age was significant for upright faces, $F(2, 93) = 17.5$, $p = .0001$, but not for upright houses. Log-transformed RTs revealed a significant main effect of age for upright houses.

In summary, the results using untransformed RTs were largely similar to the results using log-transformed RTs. All of the effects that would indicate serial processing using log-transformed RTs were replicated using untransformed RTs for upright faces. Specifically, adults showed evidence for serial processing in Dataset 1 alone and in both datasets combined. Older children also showed evidence for serial processing, but younger children did not. The main difference between the results using log-transformed and untransformed RTs were with house stimuli. However, in this case, the differences were due to marginal simple effects becoming significant (or vice versa) between log-transformed versus untransformed RTs. As shown in Figure S2, the general patterns of similarity functions across age were essentially the same.

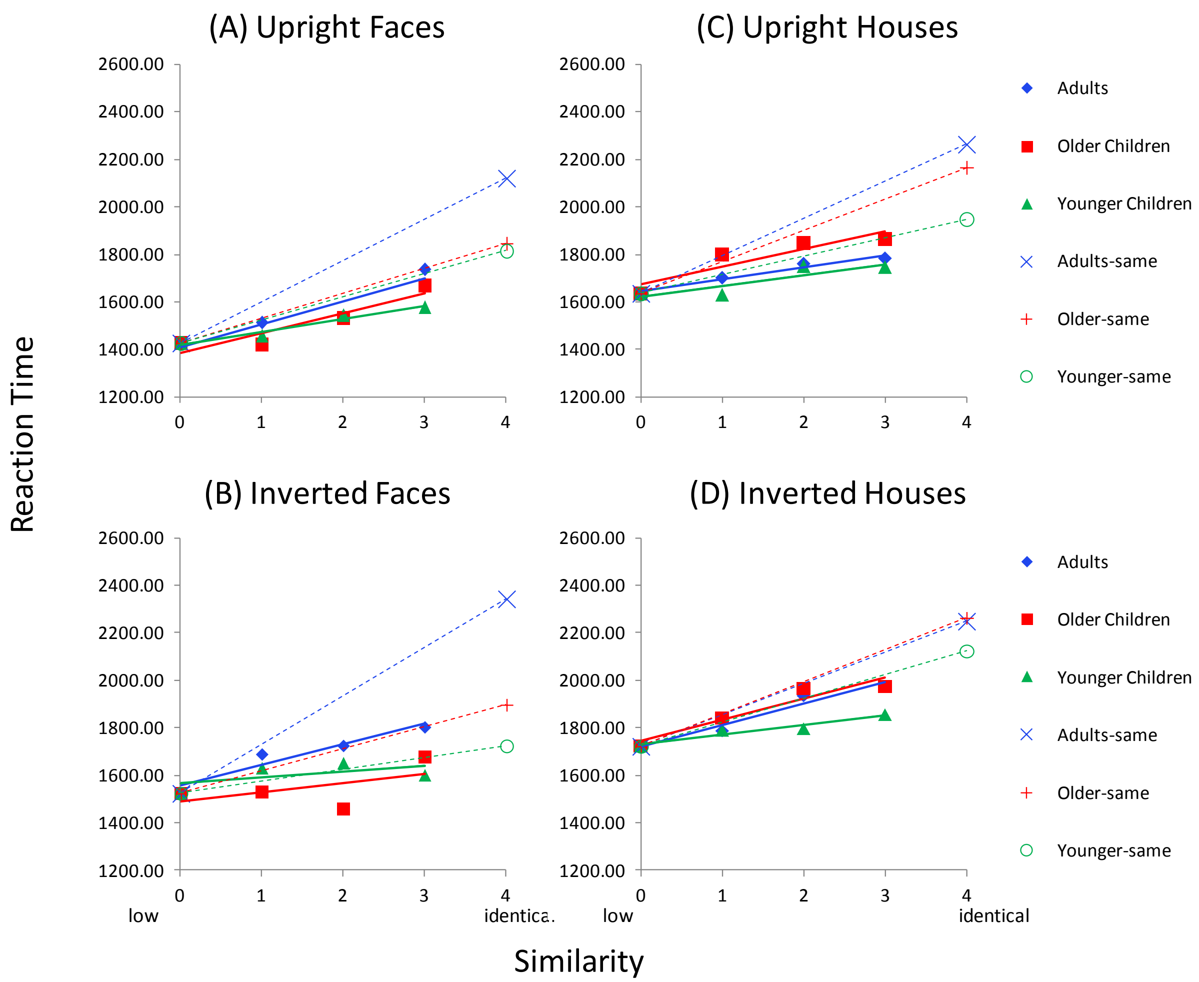

Figure S1. Face and house matching untransformed RT as a function of inversion, similarity, and age group for the featural condition. (A) Results for upright faces. (B) Results for inverted faces. (C) Results for upright houses. (D) Results for inverted houses. In each panel, similarity functions for “different” trials are indicated as solid lines and filled symbols and similarity functions for “same” trials are indicated with dotted lines and hollow symbols. In a given panel, each age group’s similarity function is scaled to the same adjusted mean value in the sim0 condition. Error bars are not shown given the complexity of the graphs.

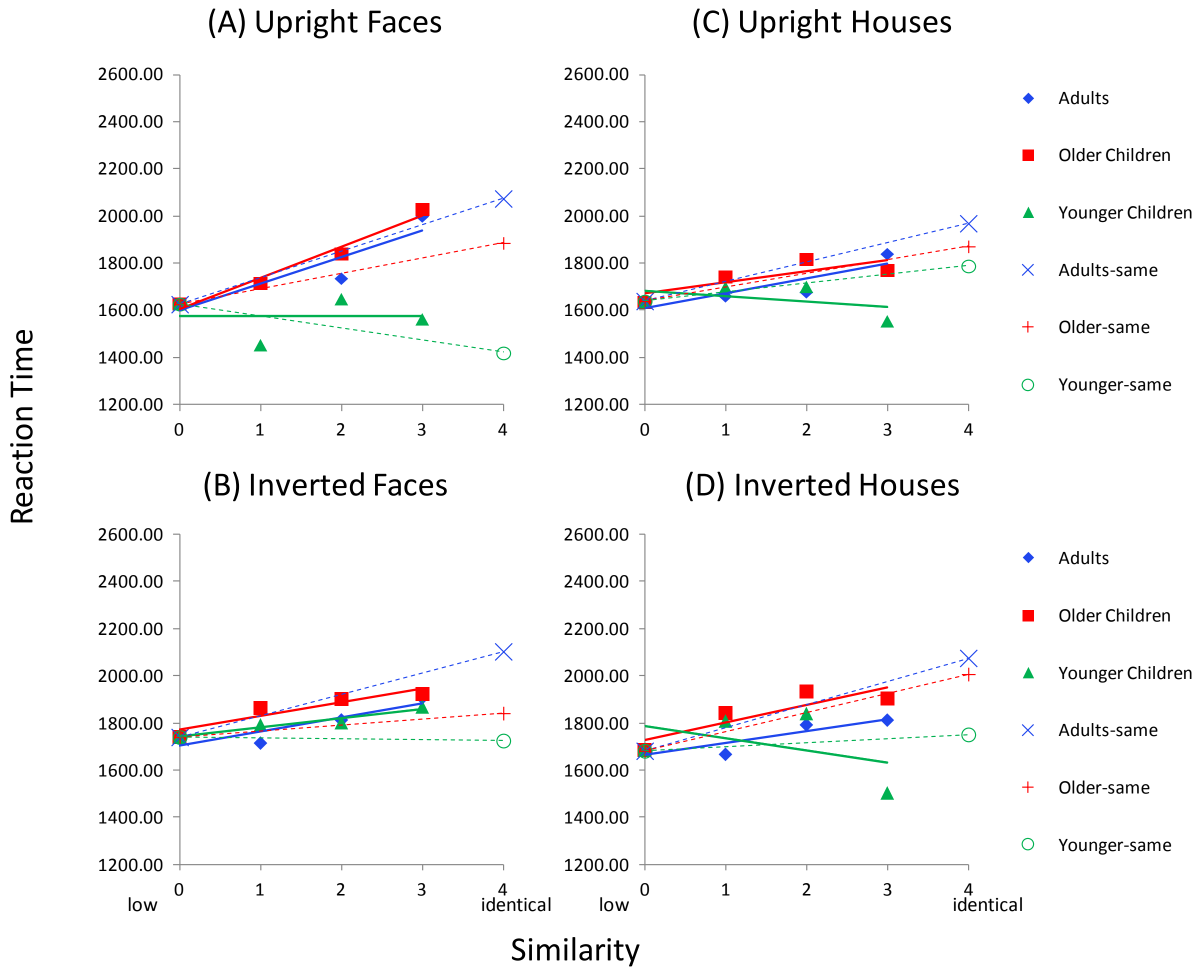

Figure S2. Face and house matching untransformed RT as a function of inversion, similarity, and age group for the 2nd order condition. (A) Results for upright faces. (B) Results for inverted faces. (C) Results for upright houses. (D) Results for inverted houses. In each panel, similarity functions for "different" trials are indicated as solid lines and filled symbols and similarity functions for "same" trials are indicated with dotted lines and hollow symbols. In a given panel, each age group's similarity function is scaled to the same adjusted mean value in the sim0 condition. Error bars are not shown given the complexity of the graphs.
